# Supplementary material for: Comparing 3D, 2.5D, and 2D Approaches to Brain Image Auto-Segmentation
Source: Bioengineering (Basel). 2023 Feb 1;10(2):181. doi: 10.3390/bioengineering10020181 (PMC9952534; doi:10.3390/bioengineering10020181)
Supplement: Supplementary file 1 [file bioengineering-10-00181-s001.zip › bioengineering-2045974-SI.pdf]

bioengineering

MDPI

Article

Comparing 3D, 2.5D, and 2D Approaches to Brain Image Auto-Segmentation

Arman Avesta [1,2,3], Sajid Hossain [2,3], MingDe Lin [1,4], Mariam Aboian [1], Harlan M. Krumholz [3,5] and Sanjay Aneja [2,3,6,*]

1 Department of Radiology and Biomedical Imaging, Yale School of Medicine, New Haven, CT 06510, USA
2 Department of Therapeutic Radiology, Yale School of Medicine, New Haven, CT 06510, USA
3 Center for Outcomes Research and Evaluation, Yale School of Medicine, New Haven, CT 06510, USA
4 Visage Imaging, Inc., San Diego, CA 92130, USA
5 Division of Cardiovascular Medicine, Yale School of Medicine, New Haven, CT 06510, USA
6 Department of Biomedical Engineering, Yale University, New Haven, CT 06510, USA
* Correspondence: sanjay.aneja@yale.edu; Tel.: 203-200-2100, Fax: 203-737-1467

Supplemental Materials

1. MRI acquisition parameters

Field strength = 3.0 tesla
Coil = 8HR Brain

Weighting = T1
Flip angle=8.0 degree
TR = 6.6 ms
TE = 2.8 ms
TI = 900.0 ms

Acquisition type = 3D
Acquisition plane = Sagittal
Matrix size = 256×256×166 pixels (X×Y×Z)
Pixel size = 1×1×1.2 mm (X×Y×Z)
Pixel spacing: along X direction = 1 mm; along Y direction= 1 mm

Copyright: © 2023 by the authors. Licensee MDPI, Basel, Switzerland. This article is an open access article distributed under the terms and conditions of the Creative Commons Attribution (CC BY) license (https://creativecommons.org/licenses/by/4.0/).

2. Comparing 3D and 2D Segmentation using the Hippocampus Dataset

We compared the 3D and 2D approaches to auto-segmentation of the anterior and posterior hippocampus using a publicly-available dataset (https://drive.google.com/drive/folders/1HqEgzS8BV2c7xYNrZdEAnrHk7osJJ--2). This comparison was done in addition to our experiments using the ADNI dataset [3] to ensure that our results hold using an external dataset. We trained 3D and 2D nnUNets over the hippocampus dataset. We performed 5-fold cross-validation for training and testing of each 3D and 2D nnUNets. The following two figures demonstrate the evolution of the

training (blue) and validation (red) losses over 1000 epochs of training, as well as the overall Dice scores (green) for the combined labels of the anterior and posterior hippocampus over the validation folds:

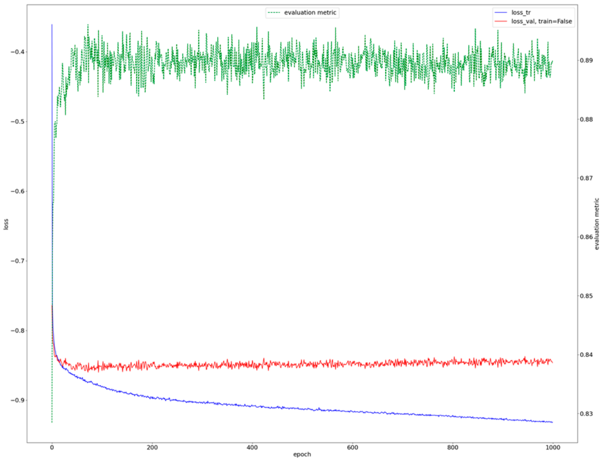

3D nnUNet

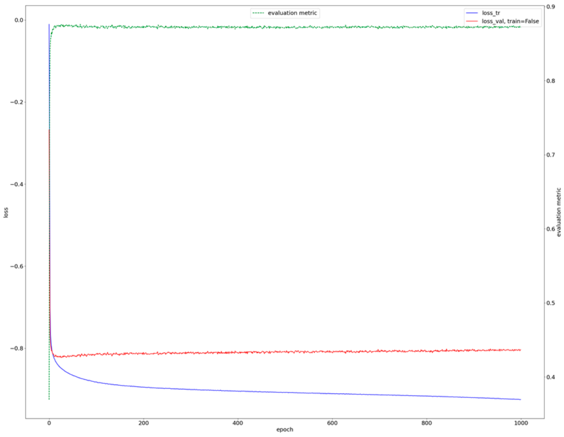

2D nnUNet

The following figure demonstrates auto-segmentation of the anterior (orange) and posterior (red) hippocampus for a single image in the validation set, done by the 3D (left) and 2D (right) nnUNet models. As seen in the figure, the 3D nnUNet achieves better Dice scores over the volume of the hippocampus in this case:

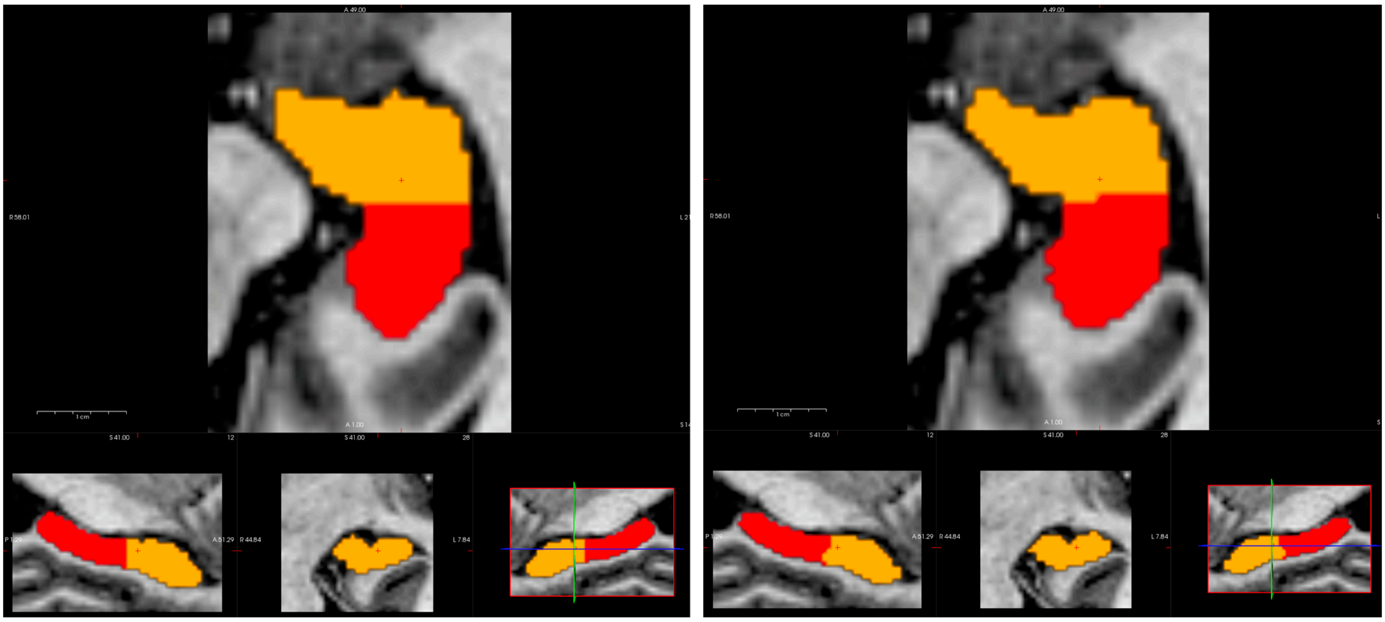

Finally, the following table demonstrates the Dice scores by 3D and 2D nnUNet models for the anterior and posterior hippocampus over the entire validation set (over 5-fold cross-validation):

	**nnUNet**	
**Brain structure**	**3D Dice (95% CI)**	**2D Dice (95% CI)**
Anterior Hippocampus	91 % (89 to 93)	88 % (87 to 89)
Posterior Hippocampus	89 % (87 to 92)	86 % (83 to 87)

The table shows that the 3D nnUNet model achieves higher Dice scores for both the anterior and posterior hippocampus. These results corroborate our results (presented in the body of the paper) that 3D models perform better in segmenting the hippocampus.

3. Pre-Processing

We corrected for intensity inhomogeneities including B1-field variations. Our pre-processing pipeline first registers the brain image to the MNI305 atlas. Then, pixel intensities are used to roughly segment the white matter. The variations in the pixel intensities in the white matter are then used to estimate the B1 field map Finally, B1 bias field correction is done by dividing the pixel intensities by the estimated bias field.[1]

The next step is the removal of the skull, face, and neck, only leaving the brain. We used a hybrid method of skull stripping that combines a watershed algorithm and a deformable surface model.[2] This method first roughly segments the white-matter based on pixel intensities. Then, watershed algorithms are used to find the gray-white matter junction and the brain surface. Next, a deformable surface model is used to model the brain surface. The curvature of the brain surface at each point is computed, and these curvatures are used to register the brain surface onto an atlas. The atlas is formed by computing the curvatures of the brain sulci and gyri in several subjects. The reconstructed brain surface, registered to the atlas, is then automatically corrected in case the curvatures in a particular region of the surface do not make sense. The resulting corrected brain surface model is used for skull stripping.[2]

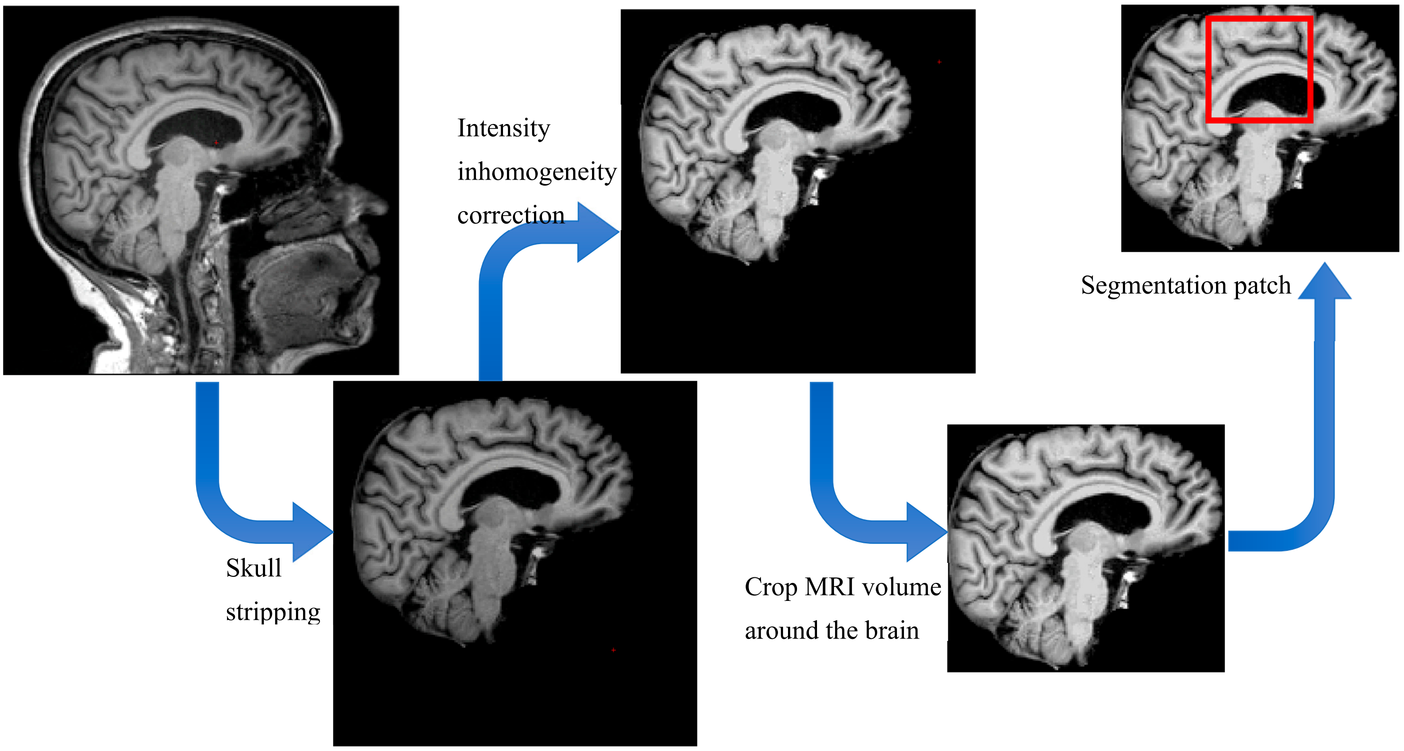

Figure S3. Pre-processing steps.

4. Segmentation Models

The architectures of capsule network (B), UNet (C), and the self-configured nnUNet (D) for 3D image segmentation are also shown. The 2D and 2.5D models had similar architectures, with the only difference that all layers of 2D models analyze 2D image slices, and the input layer of the 2.5D models accepts five consecutive image slices as input channels.

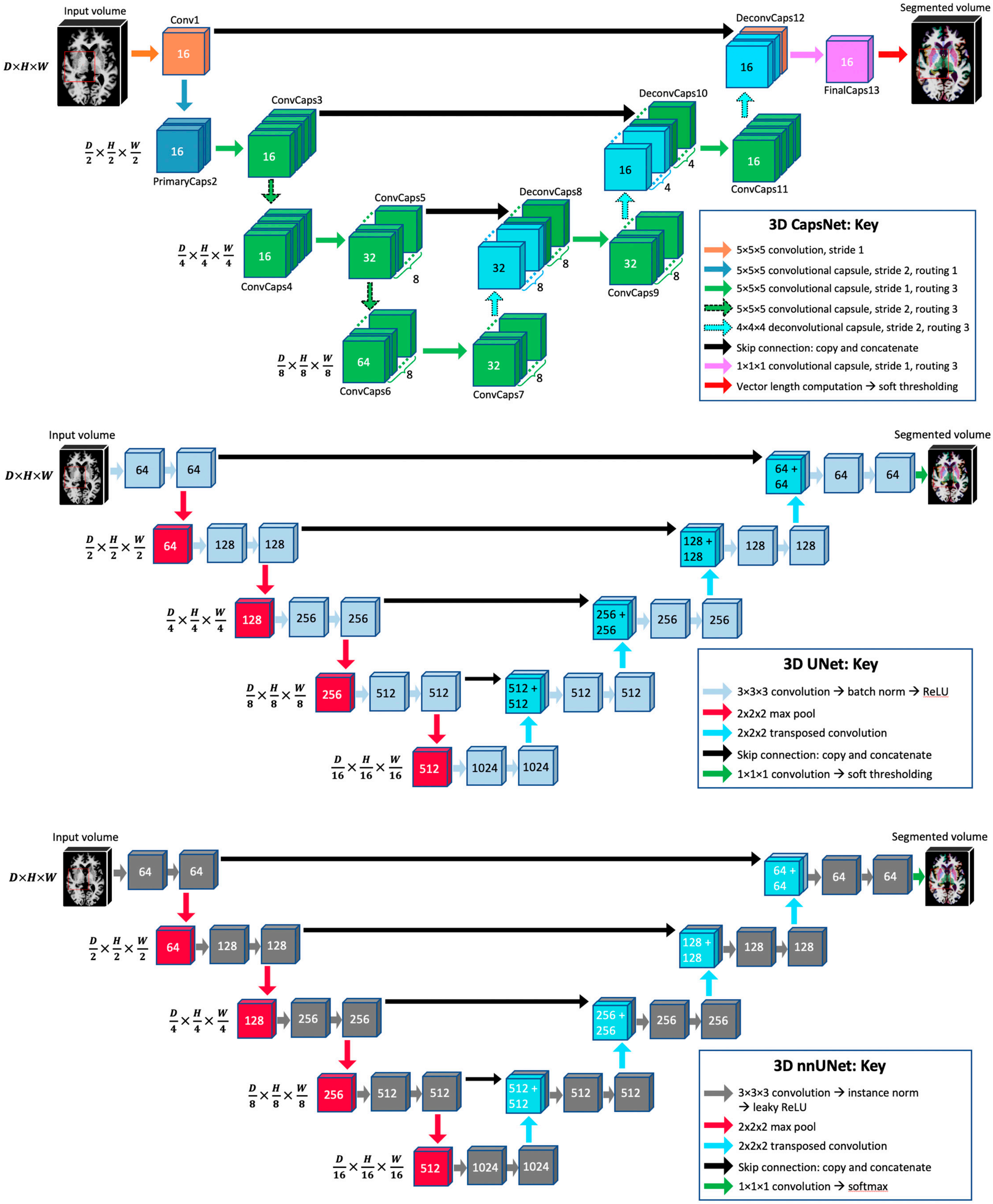
Input volume
D×H×W
Conv1
16
PrimaryCaps2
ConvCaps3
ConvCaps4
ConvCaps5
ConvCaps6
ConvCaps7
DeconvCaps8
ConvCaps9
DeconvCaps10
ConvCaps11
DeconvCaps12
FinalCaps13
Segmented volume
3D CapsNet: Key
5×5×5 convolution, stride 1
5×5×5 convolutional capsule, stride 2, routing 1
5×5×5 convolutional capsule, stride 1, routing 3
5×5×5 convolutional capsule, stride 2, routing 3
4×4×4 deconvolutional capsule, stride 2, routing 3
Skip connection: copy and concatenate
1×1×1 convolutional capsule, stride 1, routing 3
Vector length computation → soft thresholding
3D UNet: Key
3×3×3 convolution → batch norm → ReLU
2x2x2 max pool
2x2x2 transposed convolution
Skip connection: copy and concatenate
1×1×1 convolution → soft thresholding
3D nnUNet: Key
3×3×3 convolution → instance norm → leaky ReLU
2x2x2 max pool
2x2x2 transposed convolution
Skip connection: copy and concatenate
1×1×1 convolution → softmax

5. Training hyperparameters for CapsNet and UNet models

Training set size (MRI volumes): 3199
Validation set size (MRI volumes): 117
Test set size (MRI volumes): 114

Training batch size (MRI volumes): 4
Training mini-epoch size: 30 batches: during training, the validation set loss was computed after each mini-epoch

Training epochs: 50
Optimizer: Adam
Optimizer hyperparameters: $\beta_1 = 0.9,\ \beta_2 = 0.999,\ \epsilon = 10^{-8}$

Initial learning rate: 0.002
Minimal learning rate: 0.0001
Learning rate scheduling: Dynamic (via monitoring the validation set loss during training):
Learning rate was decreased by half if the validation set loss did not improve over 10 mini-epochs

6. Comparison of Total Convergence Times

Total convergence time during training was measured by the time from the start of training to the time at which the model reached the best performance on the validation set. This total convergence time includes all computations that took place during training, including saving the model after each epoch, validation after each epoch, and saving the predicted segmentations of the validation set after each epoch. The total convergence time was compared within each model (CapsNet, UNet, and nnUNet) between 3D, 2.5D, and 2D approaches.

The 3D models converged faster compared to 2.5D or 2D models. The 3D, 2.5D, and 2D CapsNets respectively converged after 46, 97, and 125 hours. The 3D, 2.5D, and 2D UNets respectively converged after 70, 95, and 130 hours. The 3D and 2D nnUNets respectively converged after 87 and 134 hours. The figure below shows these results visually:

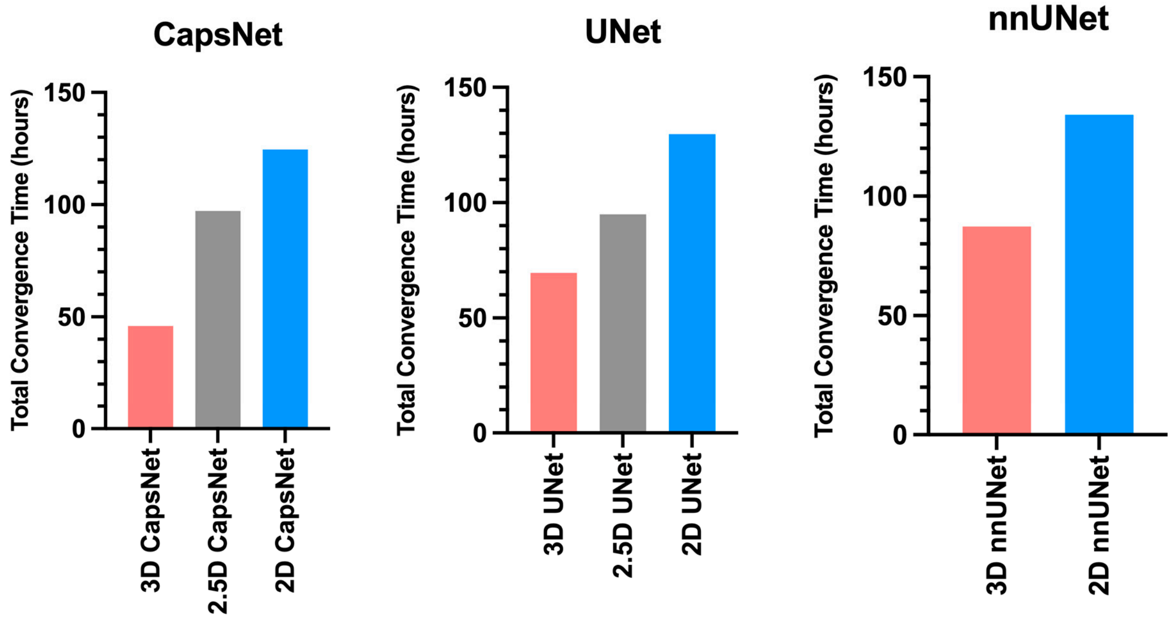

References:

1. Dale AM, Fischl B, Sereno MI. Cortical surface-based analysis: segmentation and surface reconstruction.
2. Ségonne F, Dale AM, Busa E, et al. A Hybrid Approach to the Skull Stripping Problem in MRI.
3. Weiner M, Petersen R, Aisen P. *Alzheimer's Disease Neuroimaging Initiative*. URL: https://clinicaltrials.gov/ct2/show/NCT00106899. Accessed on: 03/21/2022.; 2014.
